# Cervical dystonia and no oculomotor apraxia as new manifestation of ataxia-telangiectasia-like disorder 1 – case report and review of the literature

**DOI:** 10.3389/fneur.2023.1243535

**Published:** 2023-09-22

**Authors:** Agnieszka Bajek, Dominika Przewodowska, Dariusz Koziorowski, Maria Jędrzejowska, Stanisław Szlufik

**Affiliations:** ^1^Department of Neurology, Faculty of Health Sciences, Medical University of Warsaw, Warsaw, Poland; ^2^Genomed Health Care Center, Warsaw, Poland; ^3^Department of Neurology, Medical University of Warsaw, Warsaw, Poland

**Keywords:** ataxia-telangiectasia-like disorder 1, cervical dystonia, MRE11 gene, ataxia, case report

## Abstract

Ataxia-telangiectasia-like disorder 1 (ATLD1) is a rare neurodegenerative disorder associated with early onset ataxia and oculomotor apraxia. The genetic determination of ATLD1 is a mutation in the *MRE11* gene (meiotic recombination 11 gene), which causes DNA-double strand break repair deficits. Clinical features of patients with ATLD1 resemble those of ataxia telangiectasia (AT), with slower progression and milder presentation. Main symptoms include progressive cerebellar ataxia, oculomotor apraxia, cellular hypersensitivity to ionizing radiations. Facial dyskinesia, dystonia, dysarthria have also been reported. Here we present a 45-year old woman with cervical and facial dystonia, dysarthria and ataxia, who turned out to be the first case of ATLD without oculomotor apraxia, and with dystonia as a main manifestation of the disease. She had presented those non-specific symptoms for years, before whole exome sequencing confirmed the diagnosis.

## Introduction

Ataxia-telangiectasia-like disorder 1 (ATLD1; OMIM#604391) is an extremely rare disease with only 25 confirmed cases worldwide (1), with the largest recognized cohort in Saudi Arabia (2, 3). It is associated with early onset ataxia with oculomotor apraxia. The genetic basis of ATLD lies in a mutation in the *MRE11* (meiotic recombination 11) gene, which causes an improper response to DNA damage (4, 5). The emergence of mutations during the cell cycle is not an unusual phenomenon in healthy organisms, but a proper response against them is essential for DNA-integrity maintenance and cell survival (6, 7).

Chromosomal instability (CIN) refers to the occurrence of an increased number of faults in genetic material, leading to chromosomal rearrangements such as translocations, inversions, duplications and/or deletions (8, 9). The basis of this phenomenon is an impaired mechanism of repairing certain DNA damage. It is a characteristic feature of Chromosome Instability Syndromes (CIS) and is commonly seen in cancer cells (2, 10).

Some of the best known examples of CIS are ataxia-telangiectasia (AT) and Nijmegen breakage syndrome (NBS) (11). Although they are classified in the same group, considering their shared etiology, their clinical manifestations can be distinctly different. Recently conducted detailed genetic examination has allowed the isolation of diseases similar to them, called ataxia-telangiectasia-like disorders (ATLD) and NBS-like syndrome, which share some clinical features, but whose genetic origins turn out to be different (12).

AT syndrome (Louis-Bar syndrome) clinically presents with cerebellar ataxia (unsteadiness, lack of coordination) and cutaneous telangiectasia (numerous dilated small blood vessels) that is most apparent on the sclerae (13). Patients also suffer from immunodeficiency, radiosensitivity, cancer susceptibility, recurrent sinopulmonary infections and high levels of serum alfa-fetoprotein. AT phenotypes vary, from severe early-onset to milder adult-onset, depending on the type of mutation (14, 15).

The cause of clinical- AT symptoms is inactivation or deficiency of Ataxia-Telangiectasia-Mutated protein kinase (ATM) (16), which is one of the DNA damage response factors (DDR). ATM kinase activation is triggered by DNA double-strand breaks (DSBs) and results in phosphorylation of a variety of substrates, including p53, CHK2 and MDM2 (17), that cause DNA damage or eventually cellular apoptosis. Deficiency of ATM kinase leads to accumulation of abnormal DNA forms and disfunction of mitochondria which underlie development of AT syndrome with progressive cerebellar degeneration. This phenomenon is associated not only with pathologically increased risk of cancer development, but also prematurely aging of the body (18).

Another factor of general cellular response is MRN complex, that includes three genes (*Mre11*/*RAD50*/*NBS1*) (19) and is necessary for the process of effective DNA repair. It binds DNA ends in DSBs and activates kinase ATM monomerization (9, 17). By interaction with NBS1 protein, ATM is autophosphorilized, which is an indicator of its activation. MRN also possesses endo- and exo-nuclease activities that helps the cell to cut out and destroy defective DNA. Mutation-induced decreased levels of MRN complex components result in dysfunctions in kinase ATM actions and its accumulation at the site of DNA breakage. Moreover, deficiency of each element of MRN presents various range of symptoms.

Dysfunction of NBS1 (nibrin) protein determines the diagnosis of Nijmegen breakage syndrome (20). Neurodegeneration does not occur in NBS, but it is associated with microcephaly observed since birth, which influences craniofacial features. As in most of the CIS, the clinical picture of NBS includes immunodeficiency and an extremely high risk of developing cancer at a young age, especially of lymphoid origin (21). Other characteristic symptoms include growth inhibition, mild to moderate intellectual disability and hypogonadotropic hypogonadism in females. Prognosis is severe, due to early-onset cancers.

Nijmegen breakage syndrome-like disorder is caused by a mutation in the *RAD50* gene (22). Similarly to NBS, microcephaly, growth inhibition and intellectual disability are part of NBS-like syndrome’s clinical picture. However, it does not present with cancer predisposition, immunodeficiency and severe infections.

Although MRE11 builds a complex with NBS1, mutations throughout the *MRE11* gene (located on chromosome 11q21) do not bear any resemblance to NBS. They result in syndromes with clinical presentation more similar to AT, therefore they are called ataxia-telangiectasia-like disorder 1 (ATLD1) (5, 23). The most specific symptom of ATLD1 is progressive cerebellar ataxia as a result of neurodegeneration. Brain magnetic resonance imaging may reveal cerebellar atrophy. Characteristic symptoms of ATLD1 include oculomotor apraxia (24), slow and dysmetric saccades, delayed convergence, gaze-evoked nystagmus, radiosensitivity. Telangiectasia and increased alfa-fetoprotein levels have not been reported. Researches into cancer susceptibility are inconclusive (4). Compared to ataxia-telangiectasia syndrome, ATLD1 has later onset, milder phenotype and progresses more slowly (25).

Interestingly, not only *MRE11* gene mutation is related to development of ataxia-telangiectasia-like symptoms. Recently published data suggest that a damaged form of another molecule, called PCNA (proliferating cell nuclear antigen), leads to a disease called ataxia-telangiectasia-like disorder 2 (ATLD2) (26). PCNA is a member of the DNA sliding clamp family (27). It protects the connection of DNA with its polymerase during replication, resulting in safe and effective DNA repair (28). The main clinical features of ATLD2 include ataxia, gait instability, dysarthria, dysphagia, prelingual sensorineural hearing loss, learning difficulties and cognitive decline with age, with no immunodeficiency reported (29).

In this article we report a 45-year-old-woman who presented non-specific symptoms for a few years, was hospitalized in various facilities and was diagnosed years after the first symptoms occurred. Whole exome sequencing enabled the discovery of a pathogenic variant in the *MRE11* gene that formed the basis of diagnosis of ATLD1. This is the first case of ATLD1 that presented without oculomotor apraxia and the only one whose most distinctive symptom was dystonia.

### Case report

A 45-year-old woman was admitted to hospital for further diagnosis of speech and movement disorder, which was the reason for her repetitive hospitalization in the past. Unsettling symptoms first appeared approximately 7 years before, 2 years after the patient’s second labor. Slurred speech occurred at first and was followed by tremor of the right upper limb and periodic tremor of the left upper limb. The symptoms gradually intensified. She reported stressful situations at work a few years before, that led to her first neurological referral.

Family history of neurological disorders was negative (both parents, sister and brother healthy, two healthy children age 13, 10). Perinatal and early childhood history was uncomplicated, however she always was clumsy. The first appearance of unspecified extrapyramidal symptoms was recorded at the age of 7. For that reason she was hospitalized, but no further diagnostic steps were taken. She also reported learning difficulties as a child, however, she received higher education. At the age of 31, she underwent a left ankle stabilization procedure due to generalized ligamentous laxity (diagnosed in early childhood). She did not report any other movement system-related disorders.

Neurological examination revealed slightly dysarthric speech, cervical dystonia, dystonia of the lower part of the face, positional and terminal tremor of the upper extremities (more intensified on the right), ataxia of both upper and lower extremities and dystonic-cerebellar gait disorder. Physical examination also showed proper muscle tone and strength in all extremities, with exaggeration of deep tendon reflexes.

Her pupils were properly reactive, slight exophthalmos was observed, no sign of Kayser–Fleischer rings. No oculomotor disturbances were noted, oculography showed no signs of latency extension, no signs of decrease in amplitude as well as normal velocity of saccadic movements in horizontal direction.

Consultation with a speech therapist showed a mild dysarthria with a predominance of the cerebellar component. According to the patient, speech disorders were persistent, but their intensity fluctuated, increasing in stressful situations. The patient also complained of excessive saliva and loud swallowing. Her handwriting presented a tendency to micrography.

Although patient did not notice any difficulties in cognitive functioning, neuropsychological consultation revealed a selectively diminished cognitive function. The process of verbal material learning was slowed down, susceptibility to interference and tendency to perserveration were noted. Short-time memory was decreased. The ability to retrive information from long term memory was preserved. Difficulty in planning complex actions was noted. The efficiency of working memory, attention switching and abstract verbal reasoning skills were also decreased. Praxis, visual–spatial and verbal functions were without relevant deficits. In comparison to the previous examination, a slight exacerbation of the deficits was noted during the second neuropsychological consultation. Patient no longer reported a depressed mood, but presented a trend toward an anxious response.

In spite of slightly diminished cognitive function, the patient is professionally active and raises two children by herself.

**Figure 1:**
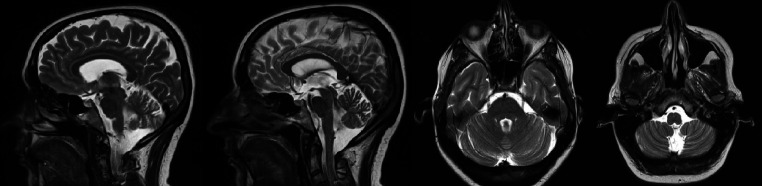
Brain magnetic resonance imaging (MRI).

MRI showed no organic brain damage that could be the cause of these symptoms. Bilaterally widened cerebellar lobes, dilated cerebellar spinal tank and mega cistera magna were noted (Figure 1). Those findings can be classified as slight cerebellar atrophy, commonly seen in ATLD1 patients.

EEG was normal. Peripheral blood smear did not demonstrate features of acanthocytosis.

Differential diagnosis included genetic conditions, such as Huntington disease, Wilson disease, neuroacathocytosis – all were excluded. Functional disorders were considered and regarded as the most probable cause of disease for years.

In the course of the current diagnosis, due to an ambiguous clinical picture, Whole Exome Sequencing was performed. WES results detected the presence of two variants in the *MRE11* gene: c.77 T > C (p.Met26Thr) and c.1090C > T (p.Arg364Ter). Both gene variants are known and described in databases (HGMD, ClinVar) as pathogenic or potentially pathogenic.

Clinical manifestations and results of molecular studies were conclusive and indicated ataxia-telangiectasia-like disorder type 1, associated with *MRE11* gene mutation, inherited in an autosomal recessive manner.

Due to escalation of cervical dystonia, the patient had 100 units of botulin toxin injected in the cervical muscles. Dopaminergic agents were used in the past, but they did not help in the management of symptoms thus medications were discontinued. Genetic counseling was offered. Due to the autosomal trait of transmission, all patient’s children are obligate heterozygotes asymptomatic carriers for MRE11 variants. Patient has got two children, who did not show any symptoms of ATLD1. In the event of reproductive planning, genetic consultation was advised. Prophylactic examinations were recommended, taking into account a lack of unequivocal data regarding cancer predominance (30). The patient was discharged in good general condition with a recommendation of further care in the outpatient clinic.

## Discussion

Chromosome Instability Syndromes can present with a variety of clinical features, with different frequency and severity, so diagnosis is often challenging. Previously mentioned diseases are the result of inherited defects in the same pathway of response to DNA damage, so it is no surprise they share similarities. In some cases the only way to differentiate genetic conditions is to perform advanced genetic tests (5), which enable the detection of precise types of variants, responsible for certain phenotypes (31).

ATLD1 is an autosomal recessive disease, therefore the presence of two pathogenic alleles is necessary to develop symptoms. The occurrence of various pathogenic variants of the MRE11 gene results in different clinical manifestations of the disease in affected individuals. Missense mutation in the MRE11 gene was associated with higher levels of MRN proteins compared with truncating mutations (3, 5).

Previously reported cases of ATLD1 showed similar type, progression and intensity of symptoms. In contrast to our patient, most of the described cases showed their first symptoms in early childhood.

Although dystonia was present in some ATLD1 patients, it is not the main symptom of ATLD1. Dystonia is classically considered a basal ganglia disease, however now it is regarded as a network disorder with involvement of the cerebellum (32, 33). Le Bar et al. characterized familial phenotype associating dystonia and cerebellar atrophy (34). Miyamoto et al. reported a patient presenting a dystonia-ataxia phenotype with severe cerebellar atrophy visible in brain MRI (35). The findings from Batla et al.’s study suggest cerebellar involvement in some cases with cervical/segmental dystonia (36). Cases of ATLD1 patients may become next evidence of the potential connection between cerebellar atrophy and dystonia.

Stewart et al. (5) reported two families with three variants of the *MRE11* gene. In the first family two cousins were homozygous for nonsense variant (c.1897C > T, R633) and in the second family two brothers shared two variants: nonsense (c.1714C > T, R571X) and missense (c.350A > G, N117S). They all presented with early-onset (1-3yo) ataxia, with oculomotor apraxia, dysarthria and cerebellar atrophy (37, 38). Progressive, distal dystonia was observed in the second family.

Fernet et al. (3) reported 10 patients from three families in Saudi Arabia from ages 5 to 37, who presented early-onset (12 months-7 years old) ataxia and oculomotor apraxia without immunodeficiency and tumor development. Dystonia was not reported. All patients were homozygous for a novel missense variant (630G > C, W210C) of the *MRE11* gene. This variant results in dysfunctional MRN complex - mutated MRE11 protein is not able to interact with NBS1 protein.

Delia et al. (39) and Palmeri et al. (40) presented a case of a pair of siblings (37and 36yo), both of who were compound heterozygotes for *MRE11* gene variants: one missense (1422C > A, T481K) and one nonsense (1714C > T, R571X) – variant also reported by Stewart et al. (5). They showed similar progression of the disease (onset at 3 and 6 years of age), both suffered from early onset ataxia, ocular apraxia and cerebellar dysarthria. Furthermore, they presented with slight dystonic movements of the hands and (in one case) of the face.

A case of a 14-year-old boy, reported in India by Mahale et al. (41), presented early-onset, cerebellar ataxia with saccade and pursuit dysfunction, choreiform movements and mild finger dystonia of both outstretched hands. Exome sequencing showed compound heterozygous variants in the *MRE11* gene (c.314 + 4_314 + 7 del; the second variant was not reported).

Raslan et al.’s (26) study showed a pair of siblings (5 and 8 yo) with two heterozygous variants in the *MRE11* gene: c.1876_1895dup (p.Lys633fs) – frameshift variant, expected to disrupt the last 48 amino acid(s) of the MRE11 protein (42); and c.1516G > T (p.Glu506Ter) – nonsense mutation, known to be pathogenic (43). Both children presented with early onset (at 2 years of age), progressive gait ataxia. The elder also showed hypotonia, choreoathetosis, oculomotor apraxia and slow saccades, mild dystonia in hands and feet, absence of deep tendon reflexes and distal amyotrophy. The brain imagining was normal.

Uchisaka et al. (30) reported a case of a severe course of ATLD in two brothers, who had the same mutation in the *MRE11* gene (c.727 T > C and g.24994G > A). The ataxic gait first appeared when they were 2 years old, progression of cerebellar ataxia and cerebellar atrophy was observed. They also had developmental delay, slurred and explosive speech, and ocular apraxia, but no dystonia or dyskinesia. At the age of 15 and 9, they were both diagnosed with stage 4 non-small-cell lung cancer with multiple bone metastases.

We report a patient who is compound heterozygote in the *MRE11* gene. One of the variants - c.77 T > C (44) is a missense mutation that causes replacement of methionine at codon 26 by threonine, an amino acid with similar properties. This variant is rarely seen - it was previously reported in an individual with dystonia (45) and in an individual with abnormalities of the nervous system (46) and is associated with breast cancer susceptibility (47). Its clinical significance is described as likely pathogenic, due to insufficient data. A second variant of the *MRE11* gene – c.1090C > T – is more frequent and known to be pathogenic. It is a nonsense mutation, resulting in absent or disrupted protein product. The coexistence of these two variants has not previously been reported in ATLD1 patients, so it may be the reason for the unique clinical picture. In comparison to other reported ATLD cases, our patient was not only older, but also did not present with the full range of symptoms. At her age the clinical picture is usually more severe. Although unspecified extrapyramidal symptoms appeared in childhood, she was relatively symptom-free up until the age of about 37 (Table 1).

**Table 1:** Clinical pictures and types of variants of some of the ATLD1 cases.

Study		MRE11 variants	Clinical picture						
			ataxia	deep tendon reflexes	facial dyskinesia	dystonia	dysarthria	ocular apraxia	cerebellar atrophy in MRI
Fernet et al. (Saudi Arabia) (3)	Family 1–4 cases	c.630G > C (p.Trp210Cys) (homozygous)	+	reduced	+	NM	NM	+	+/NA
	Family 2–3 cases		+	brisk	−	NM	NM	+	+
	Family 3–3 cases		+	reduced/ normal	−	NM	NM	+	+
Delia et al. (Italy) (39)	2 cases (siblings)	c.1442C>A (p.Thr481Lys); c.1714C>T (p.Arg572Ter)	+	reduced	+	slight dystonia of the hands/ face and hands	+	+	+
Mahale et al. (India) (41)	1 case	c.314 + 4_314 + 7 del (*as reported in the source*)	+	reduced	NM	mild finger dystonia	−	+	+
Raslan et al. (Brazil) (26)	2 cases (siblings)	c.1876_1895dup (p.Lys633fs) c.1516G > T (p.Glu506Ter)	+	absent	NM	mild dystonia in hands and feet	NM	+	−
Uchisaka et al. (Japan) (30)	2 cases (siblings)	c.727 T > C; g.24994 G > A (*as reported in the source*)	+	NM	−	−	+	+	+
Stewart et al. (United Kingdom) (5, 37, 38)	Family 1–2 cases (cousins)	c.1897C>T (p.Arg633Ter) (homozygous)	+	absent	NM	NM	+	+	+
	Family 2–2 cases (brothers)	c.350A>G (p.Asn117Ser); c.1714C>T (p.Arg572Ter)	+	normal	NM	present, distal	+	+	+
This report	1 case	c.77 T > C (p.Met26Thr); c.1090C > T (p.Arg364Ter)	+	brisk	+	severe dystonia of face and neck	+	−	+

NM, not mentioned; NA, not ascertained.

ATLD should be taken into consideration in cases of early onset ataxia with an absence of telangiectasia and a normal alfa-fetoprotein level. In the course of diagnostic procedures performed on our patient, a lot of neurological diseases were excluded. Due to the incomplete clinical picture and relatively late onset, ATLD was not considered separately for a long period of time, which may be the reason for the delayed diagnosis and the suspicion that functional disorder was the cause of symptoms.

## Data availability statement

The datasets presented in this article are not readily available because of ethical and privacy restrictions. Requests to access the datasets should be directed to the corresponding author.

## Ethics statement

Ethical review and approval were not required for the study on human participants in accordance with the local legislation and institutional requirements. Written informed consent from the patients/participants or patients/participants’ legal guardian/next of kin was not required to participate in this study in accordance with the national legislation and the institutional requirements. Written informed consent was obtained from the individual(s) for the publication of any potentially identifiable images or data included in this article.

## Author contributions

AB and SS developed the presented idea. AB wrote the paper with support from DP. DK and MJ served as scientific advisors and approved the version to be published. DK and SS supervised the project. All authors contributed to the article and approved the submitted version.
